# Interpreting Stroke-Impaired Electromyography Patterns through Explainable Artificial Intelligence

**DOI:** 10.3390/s24051392

**Published:** 2024-02-21

**Authors:** Iqram Hussain, Rafsan Jany

**Affiliations:** 1 Department of Anesthesiology, Weill Cornell Medicine, Cornell University, New York, NY 10065, USA; 2 Department of Computer Science and Engineering, Islamic University and Technology (IUT), Gazipur 1704, Bangladesh

**Keywords:** explainable AI, electromyography, stroke, SHAP, LIME, Anchors

## Abstract

Electromyography (EMG) proves invaluable myoelectric manifestation in identifying neuromuscular alterations resulting from ischemic strokes, serving as a potential marker for diagnostics of gait impairments caused by ischemia. This study aims to develop an interpretable machine learning (ML) framework capable of distinguishing between the myoelectric patterns of stroke patients and those of healthy individuals through Explainable Artificial Intelligence (XAI) techniques. The research included 48 stroke patients (average age 70.6 years, 65% male) undergoing treatment at a rehabilitation center, alongside 75 healthy adults (average age 76.3 years, 32% male) as the control group. EMG signals were recorded from wearable devices positioned on the bicep femoris and lateral gastrocnemius muscles of both lower limbs during indoor ground walking in a gait laboratory. Boosting ML techniques were deployed to identify stroke-related gait impairments using EMG gait features. Furthermore, we employed XAI techniques, such as Shapley Additive Explanations (SHAP), Local Interpretable Model-Agnostic Explanations (LIME), and Anchors to interpret the role of EMG variables in the stroke-prediction models. Among the ML models assessed, the GBoost model demonstrated the highest classification performance (AUROC: 0.94) during cross-validation with the training dataset, and it also overperformed (AUROC: 0.92, accuracy: 85.26%) when evaluated using the testing EMG dataset. Through SHAP and LIME analyses, the study identified that EMG spectral features contributing to distinguishing the stroke group from the control group were associated with the right bicep femoris and lateral gastrocnemius muscles. This interpretable EMG-based stroke prediction model holds promise as an objective tool for predicting post-stroke gait impairments. Its potential application could greatly assist in managing post-stroke rehabilitation by providing reliable EMG biomarkers and address potential gait impairment in individuals recovering from ischemic stroke.

## 1. Introduction

Stroke is a leading cause of disability and mortality, particularly among the aged population [1]. Motor disability resulting from a stroke, also known as post-stroke motor impairment, is a common consequence of a cerebrovascular accident. When a stroke occurs, it disrupts the blood supply to a specific area of the brain, leading to brain damage. This damage can affect various functions controlled by that particular region, including motor control [2,3]. The severity and specific characteristics of walking disability can vary depending on the location and extent of the brain damage caused by the stroke [4,5]. Even though there have been tremendous improvements in the treatment of post-stroke patients, most survivors still suffer functional motor issues [3]. Stroke can significantly impact an individual’s gait and walking ability [6]. Motor deficits resulting from a stroke, such as muscle weakness, spasticity, coordination problems, and balance impairments, can all contribute to alterations in gait. Hemiplegia, spasticity, foot drop, and ataxia are a few examples of post-stroke walking disabilities [7].

Electromyography (EMG) is a diagnostic procedure that measures the electrical activity produced by muscles. It can be used to evaluate and assess motor disabilities resulting from a stroke. The non-invasive muscle activity monitoring technique can detect stroke-related changes in neuromuscular coordination [8]. Post-stroke rehabilitation relies on comprehending the modified muscular attributes resulting from a stroke. EMG has significantly improved post-stroke patients’ quality of life and been pivotal in evaluating post-stroke gait recovery [9]. The frequency spectrum analysis of EMG signals aids in assessing muscle fatigue, detecting abnormal muscle activity, and studying changes in motor unit firing rates [6,10]. Mean frequency (MNF) and median frequency (MDF) are prominent and widely utilized frequency-domain features for effectively assessing muscle fatigue [11].

The artificial intelligence (AI) machine learning (ML) approach and wearable technology can be helpful techniques in a real-time physiological monitoring system for diagnostics and prognosis in everyday life and the clinical setting [12,13,14,15,16]. These technologies can significantly improve diagnostics, treatment personalization, remote monitoring, and overall healthcare management, leading to more efficient and effective healthcare services [4,12,17,18,19,20,21,22]. Although there have been previous studies on ML-based stroke-impaired gait prediction using EMG gait features, the lack of interpretability of ‘black-box’ ML models hinders implementation in clinical settings [6]. Therefore, studies are needed to investigate an EMG-based interpretable ML approach for muscular disorders in stroke-impaired gait. Explainable AI (XAI) aims to enhance the interpretability and trustworthiness of ML models [23,24,25,26,27,28]. This approach provides human-readable explanations for medical ML models, fostering trust, accountability, and fairness for clinicians. We aimed to detect muscle activity alterations due to stroke-related neuromuscular deficits using EMG measures and employed XAI to highlight EMG feature contributions in post-stroke gait predictive ML models.

Our study investigated myoelectric patterns during gait in stroke patients and healthy adults through wearable EMG sensors placed in four positions on each lower limb. To boost clinician trust in ML-based stroke-impaired gait recognition, we developed an efficient EMG-based stroke classification model. Additionally, we demonstrated EMG feature contributions and the visual interpretability of ML models using XAI frameworks like Shapley Additive Explanations (SHAP), Local Interpretable Model-Agnostic Explanations (LIME), and Anchors [26,29]. This study marks a pioneering study, emphasizing the significance of gait EMG features within ML-driven stroke prediction models utilizing XAI techniques. The main contributions of this paper can be summarized as follows:

- We introduced a comprehensive end-to-end framework integrating EMG-based machine learning and explainable AI models for predicting stroke-impaired gait patterns. Our approach utilizes data derived from a clinical experimental setup, specifically tailored for stroke prediction in real-life scenarios.
- Employing boosting machine learning algorithms, we effectively classify the gait patterns of both stroke patients and a healthy adult group by analyzing EMG spectral features. Notably, our study demonstrates an enhancement in classification performance compared to our prior report [30].
- To enhance clinical reasoning in the context of stroke-impaired gait, we employ Explainable Artificial Intelligence (XAI) methods such as SHAP, LIME, and Anchors. These methods shed light on the role of EMG variables in the stroke prediction ML models, providing valuable insights for a more nuanced understanding of the underlying mechanisms.

The study is organized into six sections. Section 2 delves into recent studies relevant to the research, while Section 3 outlines the specifics of the experimental protocol, signal processing techniques, machine learning methodologies, and explanations of the XAI methods used. The findings and outcomes are detailed in Section 4, followed by an in-depth discussion in Section 5. Lastly, Section 6 encapsulates the study’s conclusion.

## 2. Related Studies

EMG offers practical advantages as a noninvasive and quick procedure causing minimal discomfort [6]. Its clinical applications span various domains, including diagnostics, surgical interventions, personalized rehabilitation protocols, myoelectric control via biofeedback, support for clinical decisions, therapy evaluation, patient follow-ups, fatigue assessment, and even forensic medicine [31,32,33,34,35,36]. Researchers have effectively employed EMG to enhance post-stroke patients’ quality of life. Additionally, surface EMG-based machine learning has found application in gait-assistive robotics [9,37], treadmill rehabilitation [38], movement analysis for gait disorders, and recovery assessment [39]. Recent investigations have explored EMG’s potential as an alternative brain–computer interaction (BCI) for detecting movement intentions [40]. Moreover, feasibility studies have examined EMG’s use in muscle–computer interfaces [41], human–computer interfaces [42], and evaluating post-stroke gait recovery.

Survivors of strokes commonly experience diminished effort, increased muscle fatigue, and sensations of weakness when attempting voluntary force generation [10]. Understanding the underlying mechanisms driving these symptoms has spurred significant interest, with electromyography (EMG) emerging as a crucial tool in unveiling alterations within affected muscles. The comparative analysis of EMG signals from affected and unaffected muscles has unveiled indications of muscle fiber atrophy (suggestive of deficient muscle activation) [43], loss of motor units (quantified by reductions in the motor unit number index) [43], and decreased firing rates of motor units [39]. For instance, a proposed surface EMG clustering index (CI) seeks to diagnose post-stroke motor unit alterations [44]. Additionally, novel decomposition techniques for high-density sEMG recordings have been employed to assess altered post-stroke motor function between affected and unaffected muscles [45]. Studies have recorded muscle action potentials and spontaneous sEMG signals from mildly affected and unaffected muscles of stroke patients, revealing differences in myoelectric development between the two sides [38]. Another investigation demonstrated that a higher power spectrum frequency indicates stronger muscle force [46]. The median frequency (MDF) of EMG signals notably differed between affected and healthy sides, with higher values observed in the healthy side [47]. Further research discovered lower sEMG entropy in affected muscles compared to healthy muscles at similar torque levels [48]. Machine learning methodologies have been utilized to explore gait characteristics in post-stroke patients and predict stroke-impaired gait based on ground reaction force and acceleration data [4,20]. In a distinct study, ML methods were employed to categorize myoelectric patterns in stroke patients with impaired upper limbs, stroke patients without impaired upper limbs, and healthy subjects, analyzing upper limb EMG measures [49].

## 3. Materials and Methods

**Figure 1:**
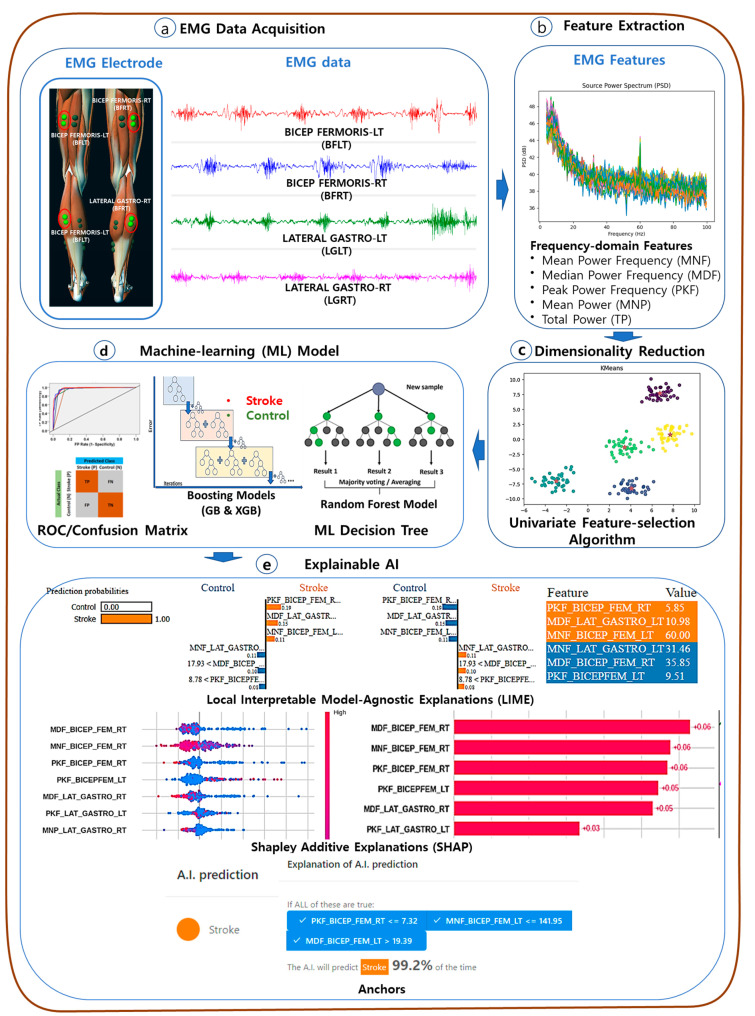
Conceptual diagram of an explainable EMG-based of stroke-impaired gait prediction model using XAI approaches. (**a**) The EMG Channel Description and sample EMG signal. (**b**) Feature extraction of EMG spectral features. (**c**) Feature reduction through feature selection approach. (**d**) Overview of various ML models with sample comparative performance matrices. (**e**) State-of-the-art explainable AI approaches (LIME, SHAP, Anchors) for interpretation of stroke prediction models.

The diagram in Figure 1 illustrates the conceptual layout of an interpretable EMG-based prediction model for stroke-impaired gait. The model incorporates state-of-the-art ML approaches and utilizes SHAP and LIME for interpretability. The methodology section of the paper provides a detailed explanation of the EMG data acquisition system, data pre-processing techniques, EMG spectral feature extraction methods, and the interpretable ML prediction models.

### 3.1. EMG Data Acquisition

In this study, the Myoresearch DTS System (Noraxon Inc., Scottsdale, AZ, USA) and Noraxon MR3 Myomuscle software were utilized for the collection of EMG data [6]. EMG data were specifically acquired on the lateral gastrocnemius and bicep femoris muscles in both lower limbs (depicted in Figure 1a). Participants refrained from consuming beverages like coffee or alcohol and from exercising before tests. Natural walking instructions were given during data collection. Figure 1a depicts raw data from the four-channel EMG setup. Additionally, the simultaneous recording of a single-channel electrocardiogram (ECG) aided in separating EMG signals from potential ECG-related artifacts.

### 3.2. Study Protocol and Cohort

This study protocol was approved by the Institutional Review Board (IRB) of Korea Research Institute of Standards and Science (KRISS), South Korea. The experiment began with participants being seated for three minutes, followed by line-following walking along a rectangular path within the experiment hall. They walked freely for about 200 m while continuous EMG data collection took place. In our study, 48 stroke patients (mean age: 72.2 years, 38% female) and 75 healthy adults (mean age: 77 years, 69% female) were included. Both groups were selected within a similar age range to minimize age-related variations in gait patterns. Stroke group participants were recruited from the Stroke Rehabilitation Center at Chungnam National University Hospital, confirmed through clinically verified MRI or CT scans. The control group comprised healthy adults without a history of stroke or underlying gait issues.

### 3.3. Pre-Processing of EMG Data

In EMG pre-processing steps, 60 Hz AC noise originating from the local power grid was removed from the EMG signal using a band-stop filter. Recorded EMG data were Butterworth Bandpass filtered, employing a high-pass filter of 15 Hz and a low-pass filter of 450 Hz. To eliminate cardiac artifacts, the FastICA algorithm was employed to denoise the raw EMG signal and isolate the ECG and motion components [50]. To eliminate low-frequency motion artifacts, which arise from the movement of cables connected to EMG sensors, a signal-to-noise ratio (SNR) was calculated for each signal by comparing raw EMG signals and the undisturbed EMG taken immediately following the muscle contraction. If the SNR fell below 18 dB, indicating insufficient signal quality, the corresponding EMG epoch was removed from the dataset [51,52].

### 3.4. EMG Feature Extraction

EMG spectral features encompass various statistical measures extracted from the power spectrum of EMG signals (Figure 1b). We employed the Welch periodogram method to derive these features [53]. This method, utilizing Fast Fourier Transforms (FFTs), calculates the power spectral density of frequency components in artifact-free EMG recordings. EMG spectral features include the mean power (MDP), total power (TP), median frequency (MDF), mean frequency (MNF), and peak frequency (PF) of the EMG waveform [54]. The MDF and MNF were evaluated according to Equations (1) and (2).

(1) $$\sum_{f=f_{0}}^{MDF(t)}PSD\left[f,t\right]=\sum_{MDF(t)}^{f_{L}}PSD\left[f,t\right]=0.5\sum_{f=f_{0}}^{f_{L}}PSD\left[f,t\right]$$

(2) $$MNF=\frac{\sum_{f=15}^{450}f_{t}PSD\left[f,t\right]}{\sum_{f=15}^{450}PSD\left[f,t\right]}$$

where PSD[f,t] is the power spectral density of the EMG time–frequency spectrum at the time instant, *t*; and *f* is the EMG frequency, ranging between *f*_0_ = 15 Hz and *f_L_* = 450 Hz.

### 3.5. Feature Selection

SelectKBest is a univariate feature selection that works by selecting the best features based on univariate statistical tests. We utilized SelectKBest, available in Scikit-learn [55], to select the most contributing EMG features according to the highest K scores, as shown in Figure 1c.

### 3.6. SMOTE for Unbalanced Dataset

The Synthetic Minority Over-sampling Technique (SMOTE) is a method that addresses class imbalance by generating synthetic instances through interpolation to create instances between the selected point and its nearby instances [56]. Since the stroke dataset is smaller than the healthy control data in this study, SMOTE was applied exclusively to the training dataset to balance the classes before testing the models on the gait EMG data.

### 3.7. Machine Learning Algorithms

In this study, ML techniques were employed to classify stroke and healthy groups based on neuromuscular responses during gait (Figure 1d). RF, a prevalent ensemble learning classifier, constructs numerous decision trees. Boosting algorithms, like GBoost and HistGBoost, iteratively combine weak learners to form robust models [57]. Experiments were conducted using Google Colaboratory, offering 16 GB RAM and a 2-core Intel Xeon Processor. ML models were implemented using the Scikit-Learn library in Python [55]. Data visualization was facilitated by Seaborn and Matplotlib libraries to enhance model performance comprehension and visually represent the outcomes [58].

### 3.8. Hyperparameter Optimization

In Gradient Boosting models, crucial hyperparameters include ‘n_estimators’ and ‘max_depth’. ‘n_estimators’ signifies the count of decision trees merged into the final ensemble model, while ‘max_depth’ determines a tree’s maximum levels during training [55]. We determined the optimal hyperparameter (a combination of ‘n_estimators’ and ‘max_depth’) via a Brute force optimization method. This method entails nested loops iterating through all possible hyperparameter combinations. Each combination is trained and evaluated on a validation set or via cross-validation, recording performance metrics like accuracy, loss, or validation error. The set with the best performance becomes the chosen optimal hyperparameter set.

### 3.9. Model Performance Evaluation Matrices

The Receiver Operating Characteristic (ROC) analysis stands as a prevalent method to evaluate binary classification model performance. The Area Under the ROC Curve (AUROC), ranging from 0 to 1, serves as a comprehensive performance indicator, with a perfect score of 1.0 denoting ideal performance. The confusion matrix, or error matrix, offers a detailed breakdown of true and false predictions. To assess model performance, we considered standard measures derived from the confusion matrix, such as accuracy, precision, recall, and F1-score. These measures offer valuable insights into the model’s prediction outcomes and can be computed using the following standard equations:

(3) $$Accuracy=\frac{TN+TP}{TN+TP+FN+FP}$$

(4) $$Precision=\frac{TP}{TP+FP}$$

(5) $$Recall=\frac{TP}{TP+FN}$$

(6) $$F1\mathrm{-score}=2\times \left\{\frac{\left(Precision*Recall\right)}{\left(Precision+Recall\right)}\right\}$$

where TP denotes the true positive, TN stands the true negative, FP represents the false positive, and FN denotes the false negative.

### 3.10. Explainable Artificial Intelligence (XAI) Approaches

#### 3.10.1. Shapley Additive Explanations (SHAP)

SHAP, an XAI technique based on game theory, delivers robust explanations for both local and global ML models [29]. It adopts an additive feature attribution approach to craft an interpretable model, representing the output as a linear combination of input variables. Using SHAP allows for the discernment of each input feature’s contribution, aiding interpretation at global and local levels [59]. This study employed Tree SHAP, tailored for boosting ML models, to elucidate the relationship between EMG features and the classification of stroke and control groups (depicted in Figure 1e). Utilizing Tree SHAP aimed to explain the significance of EMG features in distinguishing between these groups.

#### 3.10.2. Interpretable Model-Agnostic Explanations (LIME)

LIME, an open-source Explainable Artificial Intelligence (XAI) framework, serves to offer interpretability to the decision-making process of opaque machine learning (ML) models [26]. LIME defines the model explanation by the following formula:

(7) $$\xi \left(x\right)=\arg\underset{g\in G}{\min}L\left(f,g,\pi_{x}\right)+\Omega \left(g\right)$$

where G represents a set of interpretable models, and g denotes the complexity of the explanation, g∈G. Equation (7) within LIME is designed to determine an interpretable model ξ(x) by minimizing the sum of two components: the loss term L(f, g, π_x), ensuring the fidelity of the interpretable model to the black-box model, and the complexity regularization term Ω(g), which prioritizes simplicity and interpretability. LIME operates by providing explanations solely at the data level, disregarding the internal workings of the model.

#### 3.10.3. Anchor

Anchors in explainable AI (XAI) refer to a specific method designed for interpreting and explaining the predictions of machine learning models, particularly in the context of natural language processing (NLP) [27]. The Anchors method focuses on generating simple, intuitive, and human-understandable rules that capture the conditions under which a model’s prediction changes. These rules, or “anchors,” serve as concise explanations for individual predictions, making it easier for users to grasp the factors influencing the model’s output.

## 4. Results

### 4.1. Feature Selection Results

We implemented the SelectKBest approach to choose the best-correlated EMG spectral features for the binary classification of two classes, including stroke and control groups. In SelectKbest, k = 15 was selected to find top features with higher k scores.

### 4.2. Class Balance and Hyperparameter Tuning

SMOTE was implemented with default parameters (SMOTE, n_neighbors = 5) only on the training set to test the models on the gait EMG data. There are two key hyperparameters, including ‘n_estimators’ and ‘max_depth’, for the GBoost and HistGBoost models, and ‘n_estimator’ for the RF model. The optimal hyperparameter ‘n_estimators’ = 128 was selected through the Brute force optimization method for the GBoost model. Moreover, the optimal hyperparameter ‘n_estimators’ = 20 was selected through the Brute force optimization method for the RF model. On the other hand, the optimal hyperparameter ‘max_depth’ = 4 was selected through the Brute force optimization method for the HistGBoost model.

### 4.3. Performance of ML Models

The EMG feature dataset was divided into training and testing datasets, with 75% of the data assigned to the training set and the remaining 25% to the testing set. To validate the trained models, K-fold cross-validation was performed. Subsequently, the performance of the models was assessed using the testing dataset.

#### 4.3.1. Performance of Cross-Validated Model

**Figure 2:**
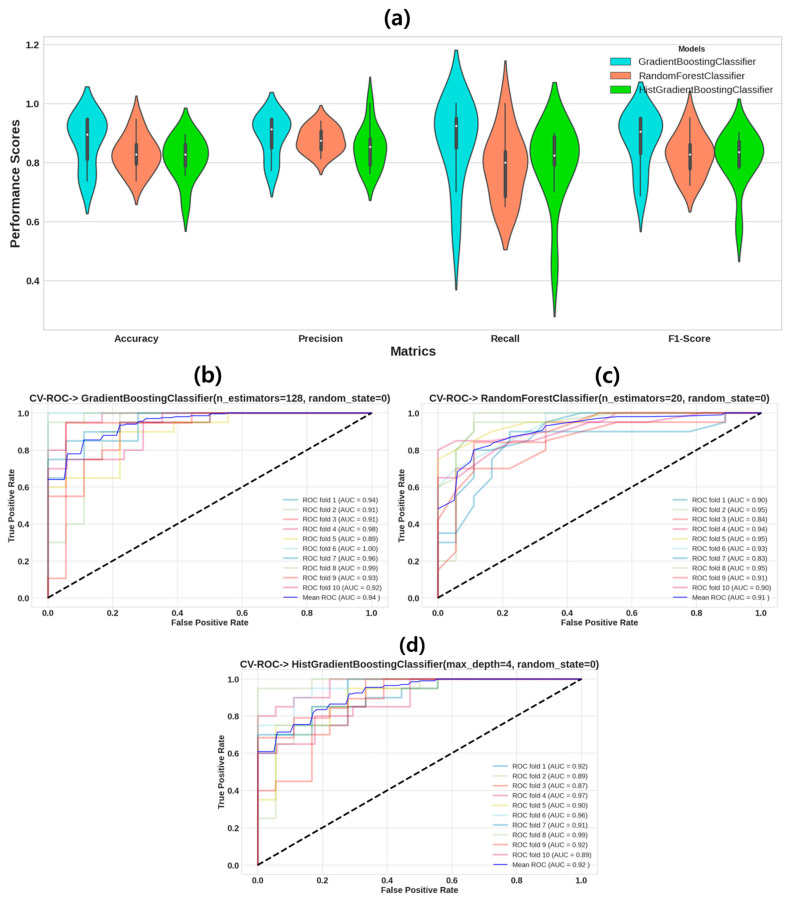
Performance parameters and Receiver Operating Characteristic (ROC) curves for k-fold (k = 10) cross-validated classification of stroke and healthy control groups using ML models. (**a**) Violin plot of performance parameters of k-fold cross-validated model for classification of stroke and healthy control groups using ML models. (**b**) Cross-validated ROC curve for Gradient Boosting (GBoost) Classifier; (**c**) cross-validated ROC curve for Random Forest (RF) classifier; (**d**) cross-validated ROC curve for Histogram Gradient Boosting (HistGBoost) Classifier. Area under ROC curve (AUC) is an indicator of prediction accuracy. The diagonal black dotted line is the reference line showing 50% accuracy.

Performance matrices of cross-validated ML models are displayed in a violin plot, as shown in Figure 2a. To mitigate overfitting, we conducted non-exhaustive K-fold cross-validation (K = 10) using the training dataset [60]. Figure 2b–d depict the ROC curves obtained from the k-fold (k = 10) cross-validation for the GBoost, RF, and HistGBoost models, respectively. The mean AUROC values are 0.94 for the GBoost model, 0.91 for RF, and 0.92 for the HistGBoost model.

#### 4.3.2. Model Performance Using the Testing Dataset

**Figure 3:**
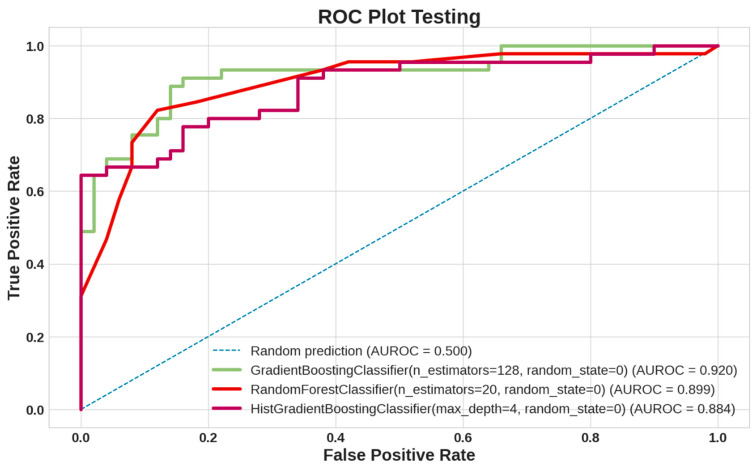
Receiver Operating Characteristic (ROC) curves for classification of stroke and healthy control groups using testing dataset. Area under ROC curve (AUC) is an indicator of prediction accuracy. The diagonal blue dotted line is the reference line showing 50% accuracy.

**Figure 4:**
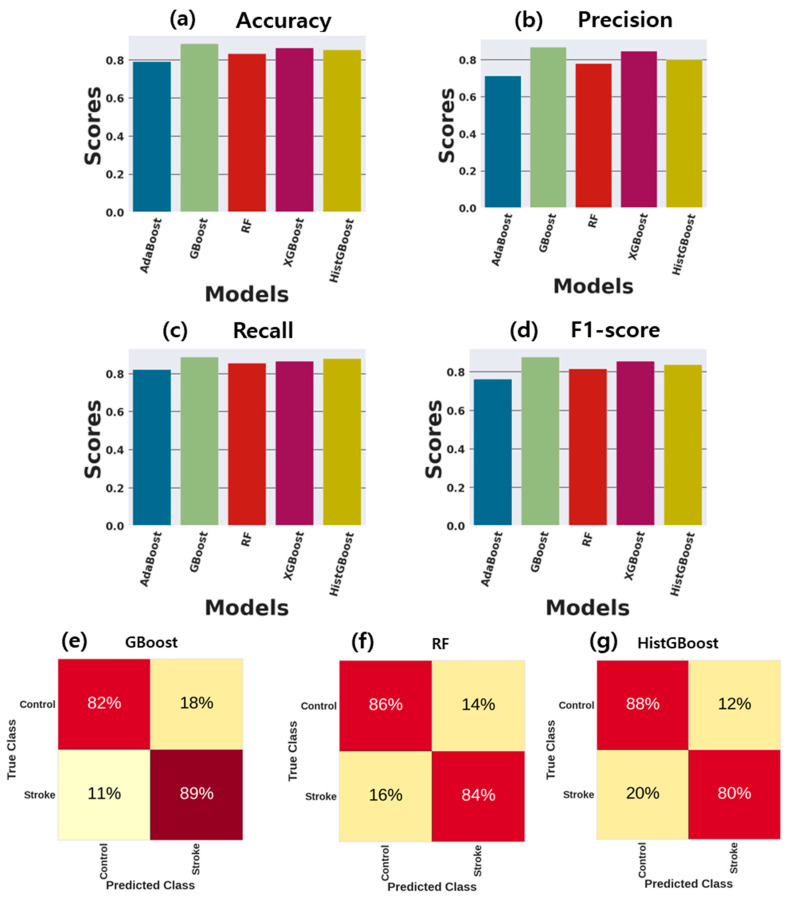
Performance matrices of ML models for classification of stroke and healthy control groups using test dataset. (**a**) Accuracy of RF and GBoost models; (**b**) precision of RF and GBoost models; (**c**) recall of RF and GBoost models; (**d**) F1-score of RF and GBoost models; (**e**) confusion matrix of test dataset for GBoost classifier; (**f**) confusion matrix of test dataset for RF classifier; (**g**) confusion matrix of test dataset for HistGBoost classifier.

Figure 3 visualizes the testing ROC curves, representing the performance curves of the GBoost, RF, and HistGBoost models using the test datasets. Figure 4a–d provide various performance measurements for the GBoost, RF, and HistGBoost models using the test datasets. The GBoost model achieved an AUROC of 0.92 and an accuracy (ACC) of 85.26%, whereas the RF model achieved an AUROC of 0.90 and an accuracy (ACC) of 85.30%. Furthermore, the GBoost and RF models demonstrated the same precision, recall, and F1-score in the classification task using the testing dataset. On the other hand, the HistGBoost model classified the testing dataset with an AUROC of 88% and an accuracy of 84.2%. It achieved the highest precision, recall, and F1-score, all at 83%, in the classification task using the testing dataset. Figure 4e–g show the confusion matrices for the GBoost, RF, and HistGBoost models using the test datasets.

### 4.4. Explainable AI Model through SHAP

In order to make our stroke-prediction ML models interpretable, the SHAP library was employed, assigning weights to EMG features to indicate their importance in the classification models. SHAP feature importance and summary plots showed the top 15 important EMG features evaluated by SHAP and their effects on the classification outcome for prediction of stroke.

#### 4.4.1. SHAP Feature Importance Plot

**Figure 5:**
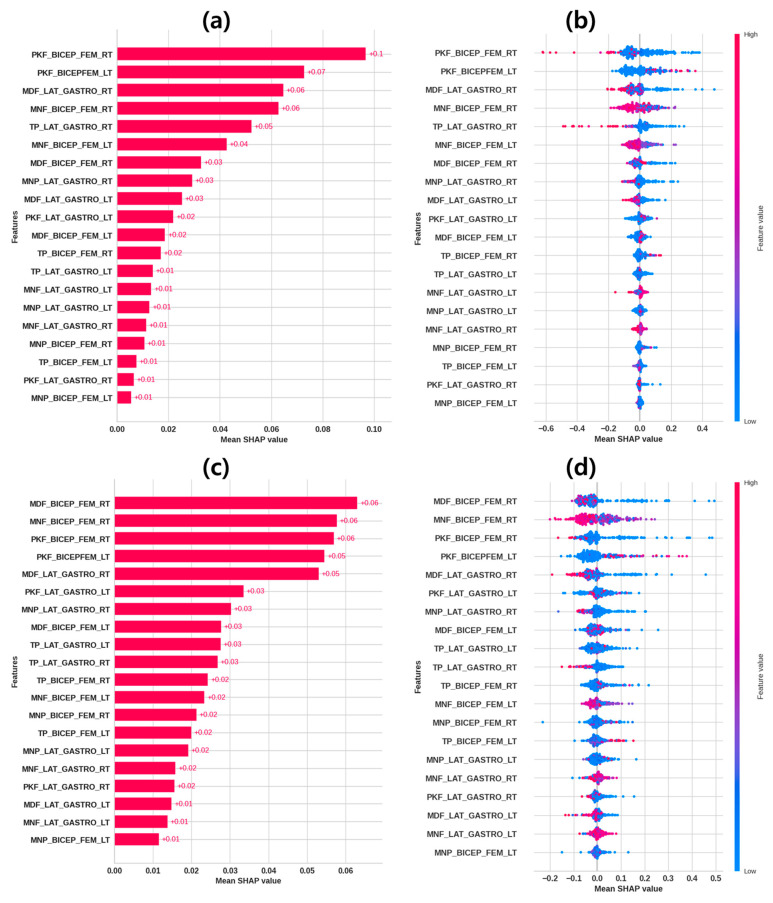
SHAP plots interpreting the contributions of EMG features in ML models for classification of stroke and healthy control groups. (**a**) SHAP feature importance plot for GBoost classifier. (**b**) SHAP summary plot for GBoost classifier. (**c**) SHAP feature importance plot for Random Forest classifier. (**d**) SHAP summary plot for Random Forest classifier.

The SHAP feature importance plot reports the mean SHAP value, describing the relative importance for each EMG feature globally. Feature importance plots of the GBoost, RF, and HistGBoost models are shown in Figure 5a,c and Figure S1a for the prediction of stroke through EMG features. Figure 5a shows that right bicep femoris and the lateral gastrocnemius muscles features (PKF_BICEP_FEM_RT, PKF_BICEP_FEM_LT, MDF_LAT_GASTRO_RT, MNF_BICEP_FEM_RT, and TP_LAT_GASTRO_RT) are among the top contributing EMG features in the GBoost model. Moreover, Figure 5c shows that right and left bicep femoris muscle features (MDF_BICEP_FEM_RT, MNF_BICEP_FEM_RT, PKF_BICEP_FEM_RT, and PKF_BICEP_FEM_LT) are among the top contributing EMG features in the RF model. Furthermore, Figure S1a shows that the right bicep femoris and lateral gastrocnemius muscle features (MDF_LAT_GASTRO_RT, TP_LAT_GASTRO_RT, PKF_BICEP_FEM_LT, PKF_BICEP_FEM_RT, and MDF_BICEP_FEM_RT) are among the top contributing EMG features in the HistGBoost model.

#### 4.4.2. SHAP Summary Plot

Figure 5b,d and Figure S1b present the SHAP summary plots illustrating the role of EMG features in prediction outcomes in classifying the stroke group and the control group. Based on Figure 5b, the lower values of PKF_BICEP_FEM_RT, MNF_BICEP_FEM_RT, TP_LAT_GASTRO_RT, and MDF_LAT_GASTRO_RT are correlated with stroke instances using the GBoost and model. Moreover, lower values of MDF_BICEP_FEM_RT and PKF_BICEP_FEM_RT indicate the stroke instances compared to control instances using the RF model, as shown in Figure 5d. Similarly, the lower values of TP_LAT_GASTRO_RT, MDF_LAT_GASTRO_RT, and PKF_BICEP_FEM_RT are correlated with stroke instances using the HistGBoost model, as shown in Figure S1b.

### 4.5. Explainable AI Model through LIME

**Figure 6:**
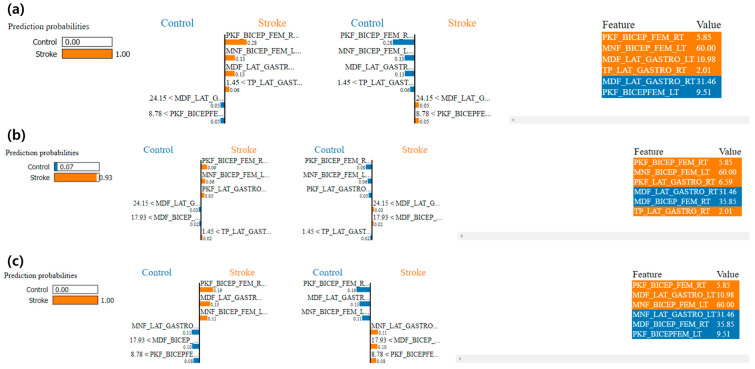
Visualization of the local contribution of EMG features through the LIME approach in classifying a single test instance (predicted class = stroke) using (**a**) Gradient Boosting (GBoost) classifier, (**b**) the Random Forest (RF) classifier; (**c**) Histogram Gradient Boosting (HistGBoost) classifier. The orange marked cells represent the features that contributed most to classifying the stroke.

Here, the LIME model was applied in ML algorithms to understand the prediction performance and individual role of EMG features in predicting stroke in gait. Figure 6 reports the LIME visualization for the GBoost, RF, and HistGBoost classifiers to forecast a local instance (predicted class: stroke). The predicted probability of that local instance of the stroke is 100%, 93%, and 100%, respectively. The most contributing features of the GBoost model are PKF_BICEP_FEM_RT, MNF_BICEP_FEM_RT, and MDF_LAT_GASTRO_RT to predict a stroke instance. Using the RF classifier, PKF_BICEP_FEM_RT, MNF_BICEP_FEM_RT, and MDF_LAT_GASTRO_RT correlate more to the prediction of a stroke instance. The most contributing features of HistGBoost model are PKF_BICEP_FEM_RT, MNF_BICEP_FEM_RT, and PKF_LAT_GASTRO_RT to predict a stroke instance.

### 4.6. Explainable AI Model through Anchors

**Figure 7:**
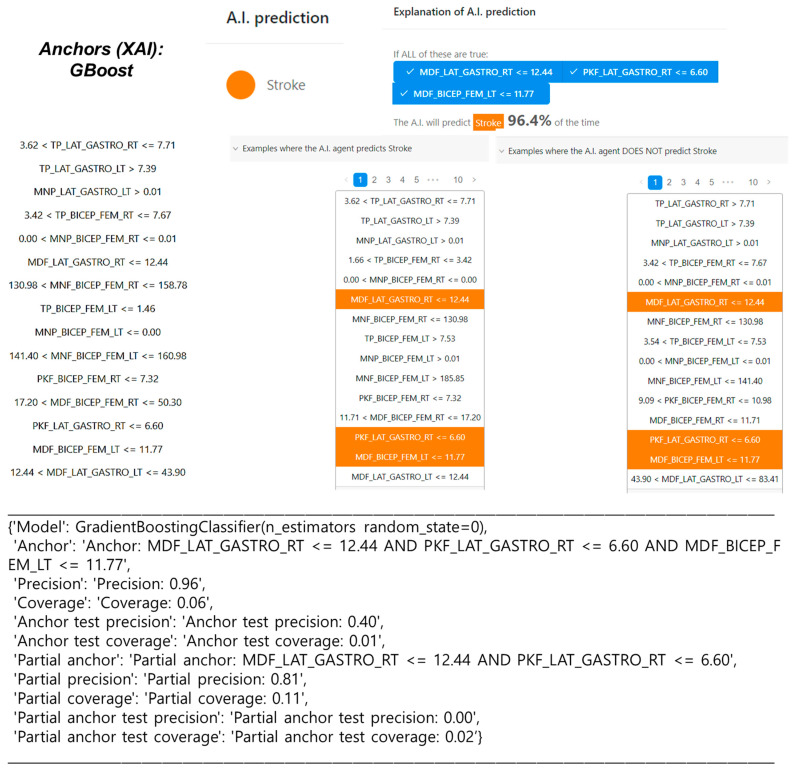
Visualization of the local contribution of EMG features through the Anchors NLP XAI approach in classifying a single test instance (predicted class = stroke) using Gradient Boosting (GBoost) classifier.

The NLP-based Anchors technique was implemented within machine learning (ML) algorithms to comprehend the prediction performance and individual contribution of electromyography (EMG) features in forecasting stroke in gait. Figure 7, Figures S2 and S3 present the Anchors visualizations for the GBoost, RF, and HistGBoost classifiers, aiming to predict a local instance with the class labeled as ‘stroke’. The predicted probabilities for that local instance of stroke are 96%, 96%, and 97%, respectively. Utilizing NLP-based XAI techniques proves valuable for non-technical users in comprehending the ML prediction model, offering a bridge for understanding the intricate details of the EMG-based stroke prediction.

## 5. Discussion

In our study, we aimed to develop a machine learning model to classify the gait of stroke patients and the healthy adult group using gait EMG data. The severity of the stroke and its impact on neuromuscular function determine the extent of muscular changes following the stroke. As a control condition, we considered the gait of healthy adults who had no history of physical muscle injuries or gait impairments, serving as a baseline for comparison.

Identifying deficits related to stroke is challenging due to the inherent heterogeneity of lower limb muscle activity during walking. The muscles in the left and right legs naturally differ from each other, further complicating the analysis. Additionally, achieving a homogeneous patient population in terms of stroke severity, lesion location in the brain, duration of post-stroke rehabilitation, and other factors poses difficulties. To assess gait impairment resulting from stroke, it is crucial to carefully select lower limb muscles for analysis. In this research, our focus was on analyzing the myoelectrical activity of the lateral gastrocnemius and bicep femoris muscles in both stroke and control groups during lower limb movements. While limited studies have explored the stiffness of the medial gastrocnemius in stroke patients, existing research has presented inconclusive findings [61]. Interestingly, both post-stroke and healthy control groups typically show higher values of median frequency (MNF) compared to mean frequency (MDF) in lower limb EMG signals [62]. This implies that the power spectrum of the EMG signal appears to be minimally impacted following a stroke.

The post-stroke patients’ gait characteristics revealed a significant change, as per the research. The study classified the gait patterns of post-stroke patients and healthy adults utilizing statistical techniques and ML methodologies. ML was utilized to predict stroke-impaired gait based on data from ground reaction force and acceleration [4,20]. In a separate study, ML methods were performed to categorize myoelectric patterns in stroke patients with impaired upper limbs, stroke patients without impaired upper limbs, and healthy subjects by analyzing upper limb EMG measures [49].

Previous research has demonstrated that a higher power spectrum frequency is indicative of stronger muscle force [46]. In contrast to the stroke group, the healthy adult group exhibited higher median frequency (MNF) values. Similar trends were observed for mean frequency (MDF) and peak frequency (PKF).

Our results illustrated that the stroke group had lower MDF and PKF values compared to the healthy adult group. These findings are supported by the study conducted by Hussain et al., which reported significant differences in mean power frequency (MNF), median power frequency (MDF), peak power frequency (PKF), and mean power (MNP) between the stroke group and the healthy control group [6]. Changes in spatial muscle activation patterns have also been associated with major alterations in muscle morphology and architecture in stroke patients compared to healthy individuals [63]. These changes may affect inter-muscular synchronization, muscle function, and overall motor function [64,65]. Despite receiving intensive post-stroke rehabilitation therapy and having no apparent temporal or spatial gait impairments, stroke patients still exhibited deficits in neuromuscular coordination due to the occurrence of stroke.

Explainable Artificial Intelligence techniques can be instrumental for clinical gait lab technicians in monitoring EMG signals to understand the severity of stroke and suggest rehabilitation plans accordingly. Technicians can gain insights into how the model accounts for patient-specific variations in most weighted EMG parameters. This study concentrated on analyzing EMG measures from specific lower limb muscle locations to evaluate stroke-induced EMG changes. While high-density EMG recordings might enhance the prediction accuracy of affected muscles, the scope here was limited to the lateral gastrocnemius and bicep femoris muscles. Participants were directed to maintain a steady pace during walking. As stroke patients received ongoing care, improving symptoms negatively impacted the accuracy of distinguishing the stroke group from the control based on EMG features. This study solely focused on power spectrum features of EMG. Future research aims to explore additional gait characteristics derived from EMG, like step length, stride length, and lower limb joint angles, potentially improving stroke and control group classification. Notably, this study exclusively investigated power spectrum features of EMG, leaving the exploration of EMG-derived gait characteristics for future inquiries.

## 6. Conclusions

This study investigated the myoelectrical activity of stroke patients and healthy adults during motor tasks, utilizing EMG analysis. Spectral power features emerged as pivotal discriminative factors, effectively explaining motor states in lower limbs between stroke patients and healthy adults. Notably, mean frequency and median frequency stood out as potential myoelectric biomarkers, distinguishing stroke patients from the healthy control group during motor tasks. Employing machine learning algorithms enabled the precise categorization of individuals into their respective groups. These findings hold significant implications for post-stroke treatment management. The identified spectral power features, alongside mean frequency and median frequency as biomarkers, offer critical insights for assessing motor function in stroke patients. Furthermore, leveraging ML algorithms for classifying individuals based on EMG features holds promise in planning effective strategies for post-stroke rehabilitation and treatment.

## Data Availability

The dataset can be obtained upon request from the corresponding author with permission from the Korea Research Institute of Standards and Science, Daejeon, Republic of Korea.

## Supplementary Materials

The following supporting information can be downloaded at: https://www.mdpi.com/article/10.3390/s24051392/s1, Figure S1: SHAP plots interpreting the contributions of EMG features in ML models for classification of stroke and healthy control groups. (a) SHAP feature importance plot for HistGBoost Classifier. (b) SHAP summary plot for HistGBoost Classifier. Figure S2: Visualization of the local contribution of EMG features through the Anchors model in classifying a single test instance (predicted class = stroke) using the Random Forest (RF) classifier. Figure S3: Visualization of the local contribution of EMG features through the Anchors model in classifying a single test instance (predicted class = stroke) using Histogram Gradient Boosting (HistGBoost) classifier.
