# Supplementary material for: Interpreting Stroke-Impaired Electromyography Patterns through Explainable Artificial Intelligence
Source: Sensors (Basel). 2024 Feb 21;24(5):1392. doi: 10.3390/s24051392 (PMC10935041; doi:10.3390/s24051392)
Supplement: Supplementary file 1 [file sensors-24-01392-s001.zip › sensors-2780709-supplementary.pdf]

Supplementary Materials

Interpreting Stroke-Impaired Electromyography Patterns through Explainable Artificial Intelligence

Iqram Hussain [1,*] and Rafsan Jany [2]

[1] Department of Anesthesiology, Weill Cornell Medicine, Cornell University, New York, NY 10065, USA
[2] Department of Computer Science and Engineering, Islamic University and Technology (IUT), Gazipur 1704, Bangladesh
* Correspondence: iqh4001@med.cornell.edu

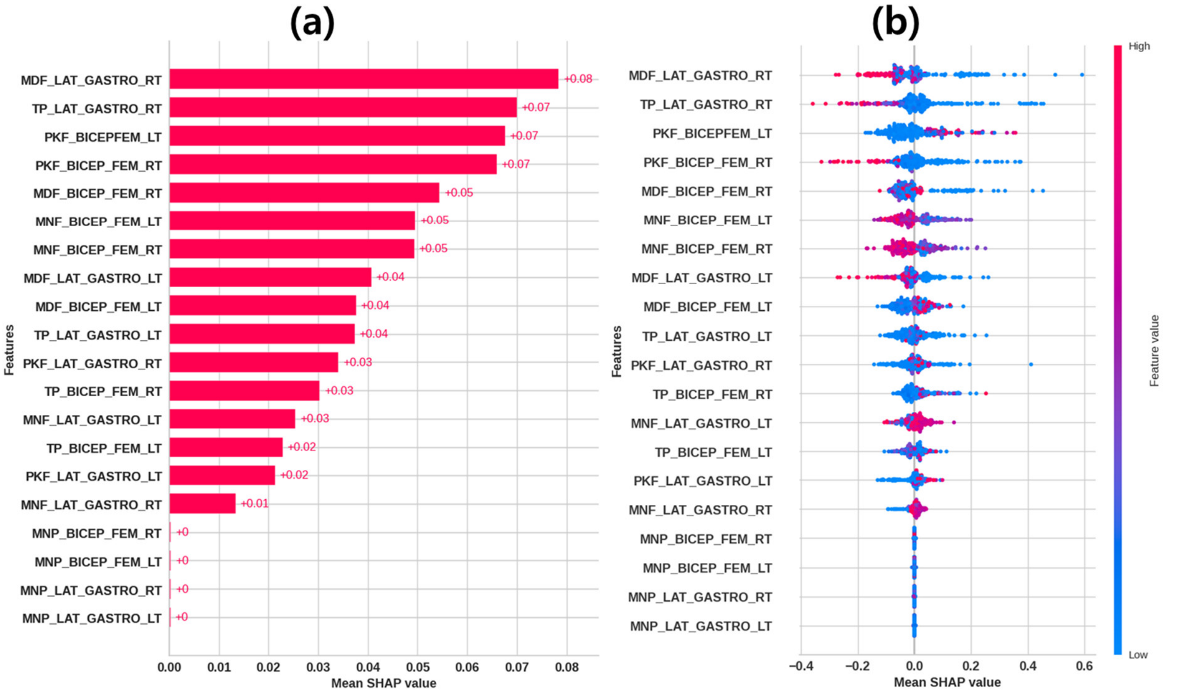

Figure S1: SHAP plots interpreting the contributions of EMG features in ML models for classification of stroke and healthy control groups. (**a**) SHAP feature importance plot for HistGBoost classifier. (**b**) SHAP summary plot for HistGBoost classifier.

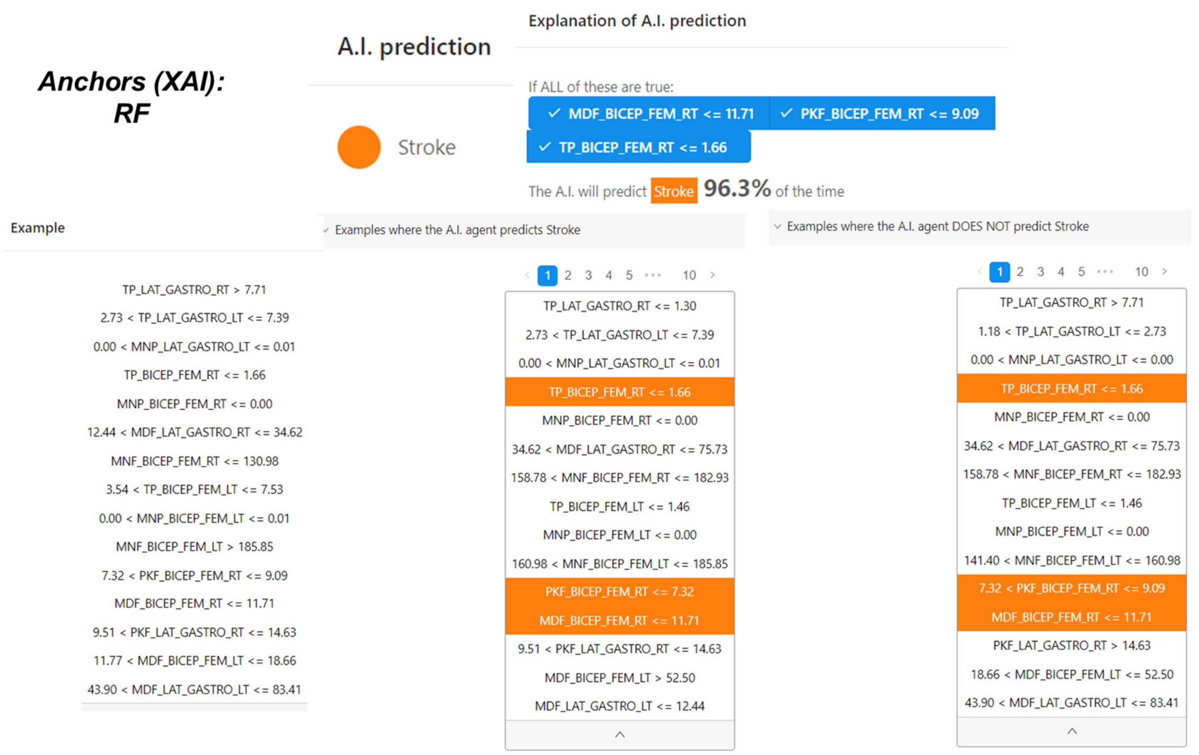

{'Model': RandomForestClassifier(n_estimators, random_state=0),
'Anchor': 'Anchor: MDF_BICEP_FEM_RT <= 11.71 AND PKF_BICEP_FEM_RT <= 9.09 AND TP_BICEP_FEM_RT <= 1.66',
'Precision': 'Precision: 0.96',
'Coverage': 'Coverage: 0.04',
'Anchor test precision': 'Anchor test precision: 0.44',
'Anchor test coverage': 'Anchor test coverage: 0.01',
'Partial anchor': 'Partial anchor: MDF_BICEP_FEM_RT <= 11.71 AND PKF_BICEP_FEM_RT <= 9.09',
'Partial precision': 'Partial precision: 0.69',
'Partial coverage': 'Partial coverage: 0.19',
'Partial anchor test precision': 'Partial anchor test precision: 0.44',
'Partial anchor test coverage': 'Partial anchor test coverage: 0.01'}

Figure S2. Visualization of the local contribution of EMG features through the Anchors model in classifying a single test instance (predicted class = stroke) using the Random Forest (RF) classifier.

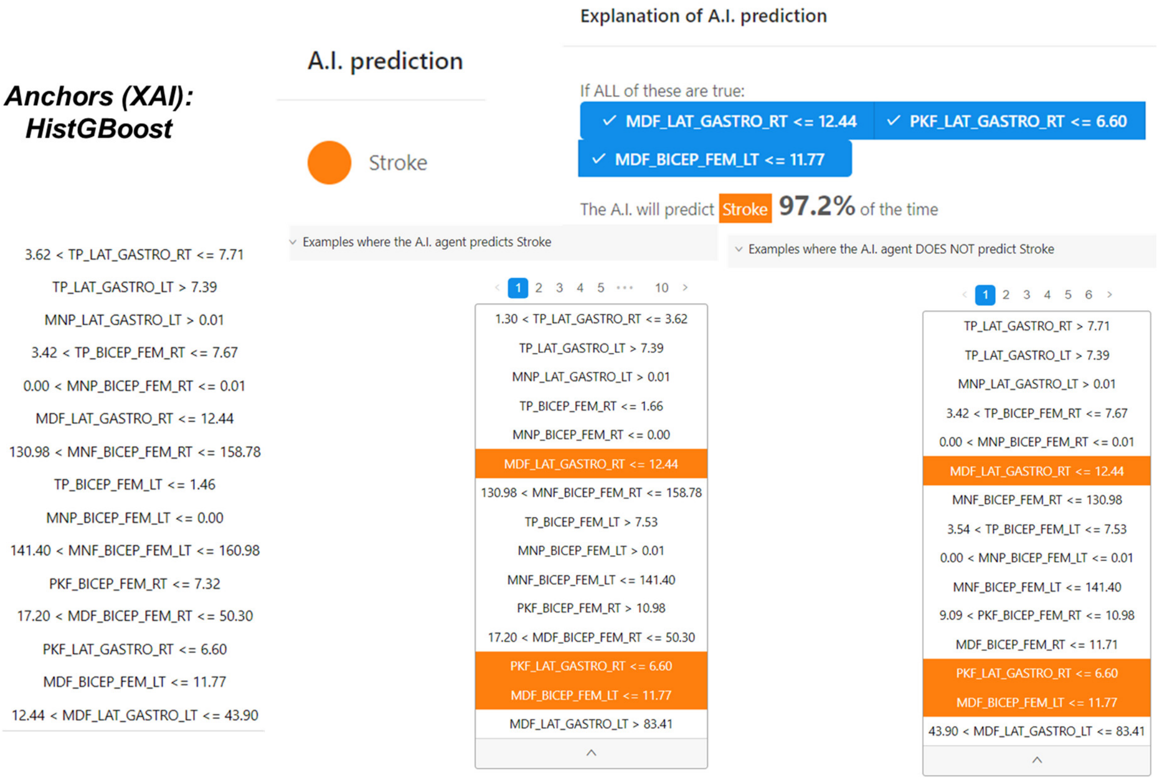

{'Model': HistGradientBoostingClassifier(max_depth random_state=0),
'Anchor': 'Anchor: MDF_LAT_GASTRO_RT <= 12.44 AND PKF_LAT_GASTRO_RT <= 6.60 AND MDF_BICEP_FEM_LT <= 11.77',
'Precision': 'Precision: 0.97',
'Coverage': 'Coverage: 0.06',
'Anchor test precision': 'Anchor test precision: 0.43',
'Anchor test coverage': 'Anchor test coverage: 0.01',
'Partial anchor': 'Partial anchor: MDF_LAT_GASTRO_RT <= 12.44 AND PKF_LAT_GASTRO_RT <= 6.60',
'Partial precision': 'Partial precision: 0.85',
'Partial coverage': 'Partial coverage: 0.11',
'Partial anchor test precision': 'Partial anchor test precision: 0.00',
'Partial anchor test coverage': 'Partial anchor test coverage: 0.02'
}

Figure S3. Visualization of the local contribution of EMG features through the Anchors model in classifying a single test instance (predicted class = stroke) using Histogram Gradient Boosting (HistGBoost) classifier.
